# Nested paradoxes around how biodiversity constrains business: Why biodiversity conservation requires paradox thinking

**DOI:** 10.1016/j.isci.2026.118128

**Published:** 2026-11-05

**Authors:** Irene Pollach, Helle Alsted Søndergaard, Mai Skjøtt Linneberg, Stefan Schaper, Anders Sanchez Barfod, Jeppe Aagaard Kristensen

**Affiliations:** 1 Department of Management, Aarhus University, 8000 Aarhus C, Denmark; 2 Department of Biology, Aarhus University, 8000 Aarhus C, Denmark; 3 Leverhulme Centre for Nature Recovery, School of Geography and the Environment, University of Oxford, Oxford OX1 3QY, UK

**Keywords:** Paradox theory, Corporate sustainability, Biodiversity offsetting, Multilevel governance, Stakeholder theory, Biodiversity footprint, Nature recovery, Mitigation hierarchy

## Abstract

Mounting evidence of nature degradation has led governmental and non-governmental actors to put pressure on companies to address their impact on biodiversity, as their economic value creation contributes directly or indirectly to biodiversity loss. However, designing effective mitigation measures requires acknowledging the complex interplay of political, social, and economic constraints that shape corporate decision-making. We elucidate how a paradox perspective provides a productive way of understanding corporate biodiversity engagement, or the absence thereof. Specifically, we illuminate how corporate biodiversity engagement is shaped by nested paradoxes operating at all levels of the economic system, wherein responses to higher-level paradoxes structurally constrain possible responses to lower-level paradoxes. We argue that meaningful corporate biodiversity engagement necessitates concerted actions across all levels, thereby empowering companies to engage with biodiversity in a proactive and consequential manner.

## Graphical abstract

**Figure:**
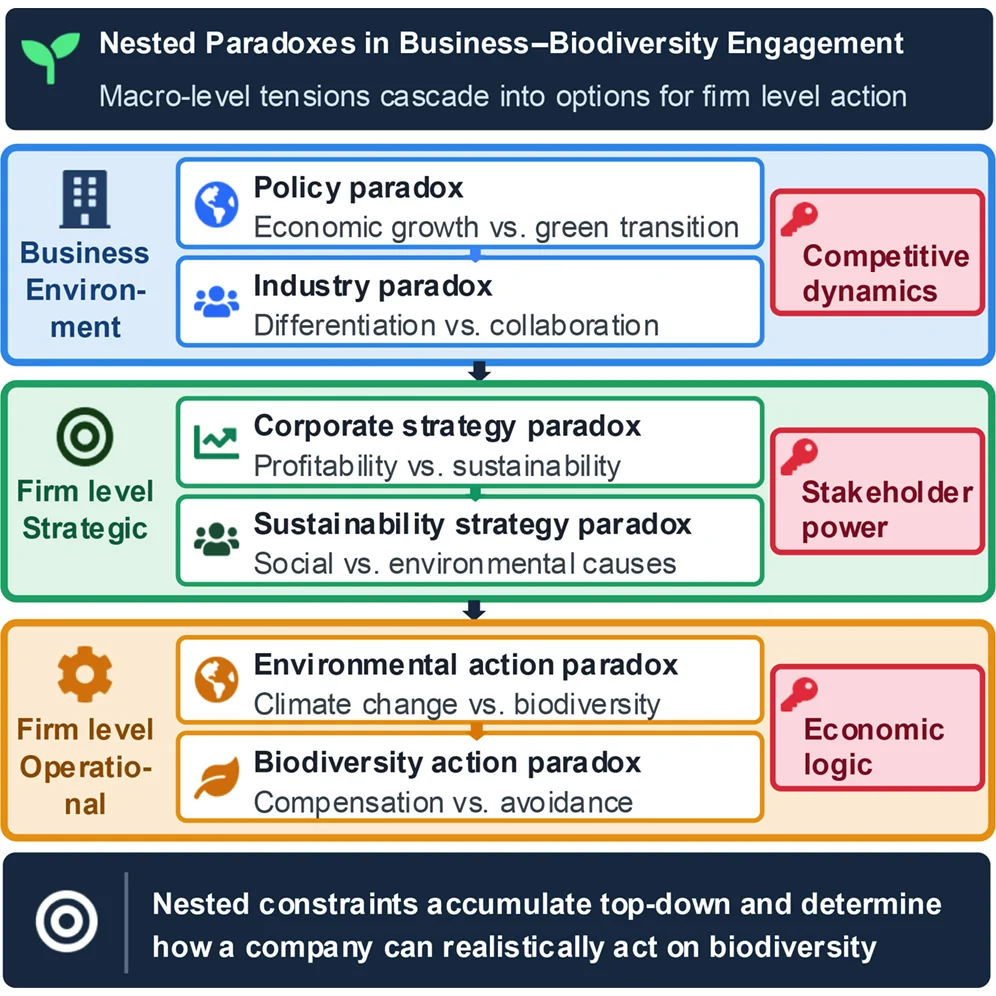

## Teaser

Ecology; Economics; Nature conservation

## Introduction

Companies consume resources and space to create value for society. In doing so, they contribute to biodiversity^1^ loss through the exploitation of natural resources, land-use conversion, and pollution.^2^^,^^3^^,^^4^ What makes negative biodiversity impact more difficult to mitigate for individual companies relative to other sustainability issues caused by economic value creation is that biodiversity losses are place-specific and can be difficult to reverse on decadal timescales.^5^ Due to the high complexity of modern supply chains and the consequent opaqueness of resource streams, it can be difficult to localize and quantify the negative biodiversity impact of companies, and hence the appropriate mitigative action.^6^

Mounting scientific evidence of nature degradation has motivated policymaking at all levels to put biodiversity loss on the public agenda.^7^^,^^8^^,^^9^^,^^10^ Especially the 2022 Kunming-Montreal Global Biodiversity Framework (GBF) with its 23 overall targets for 2030 sets a formalized global agenda for biodiversity conservation.^11^ The GBF explicitly acknowledges the role of companies and calls for policy measures that require businesses to mitigate their impact on biodiversity.^12^ In parallel, the EU has adopted a Biodiversity Strategy for 2030 and subsequently passed the accompanying Nature Restoration Law, aimed at restoring degraded ecosystems.^13^^,^^14^ In addition, the EU Corporate Sustainability Reporting Directive (CSRD) was passed in 2024, requiring companies that fulfill certain criteria to report on their environmental and social impact, including biodiversity.^15^ However, these reporting requirements were later simplified to alleviate the pressure on companies to dedicate resources to extensive reporting infrastructures.^16^^,^^17^

Despite these highly visible policy efforts, the integrity of our biosphere remains one of the most severely transgressed planetary boundaries that would be essential for staying in a safe operating space for humanity.^18^ In this article, we adopt an interdisciplinary approach, synthesizing insights from biology and business, to provide a holistic and more realistic account of how and why companies are constrained in implementing more substantive biodiversity conservation measures. Applying a paradox lens to the business-biodiversity conundrum, we argue that corporate biodiversity conservation is situated at the lowest level in a hierarchy of nested paradoxes that span all levels of the economic system. In this hierarchy, responses to higher-level paradoxes limit the range of possible responses for lower-level paradoxes. We consequently argue that effective policy intervention requires the explicit incorporation of paradox management strategies at all levels as a prerequisite for corporate biodiversity engagement.

## What are paradoxes?

Paradoxes are integral elements of complex systems, as different elements within such systems inevitably create tensions with other elements.^19^^,^^20^ The core characteristic of a paradox is a perceived persistent conflict between two oppositional demands, for which an either-or solution is not a viable option. While the tensions resulting from a paradox can never be truly resolved, they can remain dormant, thus not requiring any action, until external conditions make a dormant paradox salient.^19^^,^^21^^,^^22^ These conditions include a plurality of stakeholders with contradictory views and different power bases; limited temporal, financial, or natural resources; and ongoing contextual changes.^19^

For companies, paradoxes are a natural element of doing business, as they constantly need to address conflicting but equally legitimate stakeholder demands under resource constraints, which require compromises, trade-offs, and optimization decisions.^19^^,^^23^^,^^24^^,^^25^^,^^26^ Paradoxes experienced by companies do not necessarily exist in isolation, but can also be intertwined, knotted, or nested.^19^^,^^27^^,^^28^ They can also cascade from the organization level to the individual level and vice versa.^19^ Further, organizational paradoxes can be embedded in larger paradoxes at the system level but have been rarely examined as such in previous research.^20^^,^^29^

Individuals and organizations that are faced with a paradox typically respond with the generic strategies^19^^,^^21^^,^^30^^,^^31^^,^^32^^,^^33^ summarized in Table 1.

**Table 1:** Generic paradox management strategies

Strategy	Explanation	Response type
Denial	Talking a paradox into non-existence	Defensive
Spatial separation	Compartmentalizing the oppositional demands physically, structurally, or functionally	Defensive
Temporal separation	Addressing one demand of the paradox now, while deferring responses to the other demand to the future	Defensive
Acceptance	Acknowledging the oppositional demands, but ignoring them, as long as they do not require immediate action	Proactive
Synthesis	Embracing the oppositional elements with a “both-and” approach that requires learning, innovation, and change	Proactive

Organizations may be able to treat paradoxes successfully as optimization problems through a cost-benefit analysis, as long as the paradox remains dormant.^34^ However, if and when the paradox becomes salient through stakeholder demands or resource scarcity, conflicts and struggles will surface,^31^ since either none of the two oppositional demands of a paradox has been addressed (*Denial* and *Acceptance*) or the two oppositional demands have been dealt with separately (*Spatial and Temporal* S*eparation*). While *Denial* and *Separation* are considered defensive strategies intended to suppress contradictions or minimize their salience, *Acceptance* and *Synthesis* are more proactive in nature, embracing the contradictions as natural. The *Synthesis* approach is the only strategy that works productively with the paradox, since it goes beyond routine solutions,^35^ but has been criticized for being unfeasible and too idealistic, especially for complex, system-level paradoxes.^29^^,^^36^

## Understanding paradoxes around biodiversity and business

While companies are undoubtedly causal agents of biodiversity loss, attributing direct responsibility to companies fails to acknowledge how paradoxes in economic value creation constrain companies’ abilities to engage in biodiversity conservation. We adopt the division of the economic system into macro, meso, and micro levels to arrive at a more granular understanding of corporate biodiversity engagement or the absence thereof. The macrolevel of the economic system includes national and international policymaking, which shapes business activities through regulation, taxes, subsidies, or incentives. The meso level captures how market forces shape competition and collective action within an industry. Lastly, the microlevel encompasses the dynamics within individual companies. These three levels of the economic system constitute interrelated sub-systems that depend on one another to create economic value.^37^^,^^38^ Efforts to mitigate the negative effects of this value creation are typically made at all three levels, as is the case for biodiversity conservation, which is on the agenda of policymakers, industry associations, and companies. Adding a paradox lens onto this multilevel system enables us to identify tensions between economic value creation and biodiversity across the three levels and to elucidate how they exert a direct or indirect influence on biodiversity conservation actions or a lack thereof.

### The macro level

In the broader business environment, policymakers across all levels (UN, EU, national) constantly need to navigate between the contradictory goals of stimulating economic growth and paving the way for a green transition, both of which are high on all political agendas.^39^^,^^40^^,^^41^ This *policy paradox* is reflected, for example, in the UN Sustainable Development Goals, which aim to increase social development as well as to reduce environmental degradation. These goals are often contradictory, as social development typically leads to increased resource use and waste production, at least temporarily, thereby challenging environmental sustainability.^42^ The policy paradox becomes particularly salient in times of recession and geopolitical conflicts, where rekindling economic growth and independence is a top priority on political agendas^43^^,^^44^ at the expense of sustainability agendas. As one outcome of such efforts, the EU has recently relaxed its sustainability and supply chain reporting guidelines for European companies to increase their competitiveness vis-à-vis U.S. and Chinese companies, which are not subject to such regulations.^45^^,^^46^ Betting on “green growth” rather than degrowth, the “green economy” discourse adopted by national and international policymakers typically denies the paradox between economic growth and environmental protection and may protect the economy more than nature, as critics argue.^47^^,^^48^^,^^49^

Especially the decarbonization transition toward solar power and battery-driven vehicles increases the demand for minerals, such as aluminum, lithium, copper, cobalt, and nickel. This growing demand threatens biodiversity through an expansion of metal mining and refinement activities, coupled with the challenge of disposing of and recycling solar-panel and battery waste.^50^^,^^51^^,^^52^ Conversely, all else being equal, countries with high sustainability ambitions may need to accept the risk of an immediate loss of competitiveness, at least in some sectors, for the sake of the green transition. A current example is the introduction of a CO_2_ tax in the Danish dairy sector, which farmers fear will hurt the international competitiveness of their products.^53^^,^^54^ How policymakers navigate the policy paradox, i.e., whether they manage to incentivize pro-environmental behavior and dis-incentivize detrimental behavior or merely adopt a “green economy” discourse that denies the paradox has implications for companies’ global competitiveness as well as for the natural environment and therefore ultimately biodiversity conservation.

### The meso level

Companies within the same industry typically experience contradictory tensions between competition and cooperation. To address any industry-wide problems effectively, companies would need to collaborate in order to lower innovation costs and minimize risks, yet market forces incentivize them to protect their own competitive advantage and seek individual solutions instead.^19^^,^^55^^,^^56^^,^^57^ Environmental sustainability intensifies this paradox, because many ecological problems are collective problems that exceed the capabilities of individual firms.^58^ Companies therefore face a persistent tension between collectively addressing sustainability issuesand competing against each other for market share, reputation, or innovation leadership.^59^^,^^60^

In this paradox, competition is typically the more powerful pole, leading companies to pursue sustainability solutions individually, if at all.^59^ Especially when companies use environmental initiatives for brand building, a collaboration with a close competitor can endanger their competitive advantage. For example, fast-fashion giant H&M has attempted to brand itself as the most sustainable fast fashion company for years^61^^,^^62^^,^^63^–despite greenwashing lawsuits^64^^,^^65^^,^^66^^,^^67^^,^^68^–rather than joining forces with industry peers to enforce common systems and standards for textile sourcing, use, and recycling. Meanwhile, in industries where companies do not use environmental sustainability as a competitive differentiator, collaborations in the realm of sustainability are more likely. For example, Coca-Cola and Pepsi are fierce competitors in the bottled beverage industry when it comes to brand images, but joined forces in plastic-bottle recycling,^69^ which is a non-differentiating factor in this industry. They were, however, later sued for having overstated the recycling potential of plastic bottles.^70^

With growing pressure on companies to measure, quantify, and report on their environmental impacts, and a lack of binding industry commitments, one can be reasonably concerned that companies will set more focus on competition than collaboration in order to generate an economic return on their investments in environmental sustainability. Thus, industry competition can work as an obstacle against corporate sustainability efforts, including biodiversity conservation, as such efforts lack the potential to create competitive advantage for companies in some industries.

### The micro level

The micro level of corporate action typically consists of hierarchically organized sub-levels, including the strategic and the operational level.^71^ In relation to biodiversity, these include the overall corporate strategy and the corporate sustainability strategy at the strategic level and corporate environmental action and corporate biodiversity action at the operational level. In the following, we examine paradoxes around business and biodiversity at each of these sub-levels.

### Corporate strategy

Companies must navigate between the incompatible demands of multiple corporate stakeholder groups in their strategy making,^21^ resulting in a *corporate strategy paradox*. At the core of this paradox lies the persistent contradiction between demands for various forms of sustainability and demands for profitability from owners and current and future shareholders,^21^^,^^29^^,^^72^^,^^73^ including high social standards and living wages (employees and supply chains), high-quality products (consumers), and planetary health (society at large and nature).^74^^,^^75^^,^^76^ All these demands produce desirable outcomes, yet companies cannot satisfy these competing demands simultaneously due to persistent resource constraints.^26^ Companies therefore need to prioritize the demands of those stakeholder groups that can threaten the existence of the organization, while disregarding the demands of less powerful stakeholders.^25^^,^^77^ These power dynamics may lead to paradox response strategies in favor of one powerful stakeholder group rather than societal interests.^26^^,^^78^

Shareholders are a stakeholder group that is legally empowered to influence a company’s strategic decisions and can push companies toward neglecting demands for planetary health, if this conflicts with their own immediate interest in short-term profit maximization. A case in point is British Petroleum (BP), which recently shifted its strategy from renewable energy back to oil and gas, after shareholders called for higher profits.^79^ Conversely, shareholders can also demand more sustainability actions, if they are willing to forgo short-term profits. For example, shareholders of sportswear giant Nike have demanded more climate action and greater supply chain transparency.^80^

Individuals with multiple stakeholder roles can also encounter paradoxical tensions between their own conflicting demands. For example, as members of society, individuals may expect high environmental standards from companies, while at the same time being price conscious in their roles as consumers due to financial constraints.^81^^,^^82^^,^^83^ If consumers “vote with their wallets” for cheaper, less sustainable products, they simultaneously disempower society as a corporate stakeholder and its demands for long-term planetary health.

Both consumer choices and shareholder demands can thus make it necessary and legitimate for companies to prioritize economic goals at the expense of goals that could lower their negative environmental impact. Even though nature is treated as a corporate stakeholder in its own right in economic scholarship^84^^,^^85^^,^^86^^,^^87^ and recent EU regulation (e.g., CSRD, EU 2022/2464), it has neither inherent power nor a voice of its own in interactions with companies.^88^^,^^89^ For nature to become a more powerful stakeholder in corporate strategy making, other powerful stakeholder groups, such as shareholders or consumers, would need to speak on its behalf,^86^^,^^87^ or companies themselves would need to recognize their continued dependence on planetary ecosystems as a strategic priority for their long-term survival.

### Sustainability strategy

When devising their sustainability strategy based on the overall corporate strategy, companies need to navigate between satisfying stakeholder demands for environmental protection and stakeholder demands for social concerns. Social sustainability is closely connected to wage levels and terms of employment in companies and their supply chain, thus having financial implications for companies, similar to efforts in the realm of environmental protection.^23^^,^^73^^,^^74^ Companies can never fulfill all social and environmental demands in full due to resource constraints and diverging stakeholder interests within sustainability. What exacerbates this paradox is that companies can face situations in which prioritizing environmental problems worsens social problems and vice versa. For example, closing a production facility in a country with low environmental standards to avoid environmental problems in a company’s supply chain is likely to damage local livelihoods and bring social hardship to the affected area. Conversely, creating employment, better infrastructure, and improved living conditions in underdeveloped regions may harm local ecosystems due to increased resource use and pollution.

While companies may attempt to address both poles of the paradox to some degree, they need to temporally separate different sustainability issues according to their resource intensity due to resource constraints. Companies will predictably gravitate toward less demanding sustainability efforts that provide them with immediate benefits, for example. in the form of public visibility, while deferring more resource-intensive solutions to the future.^73^^,^^90^ This dynamic may systematically disadvantage biodiversity conservation in corporate paradox management, as the long-term character of ecological change provides minimal immediate gains for companies or society at large.

### Environmental action

At the operational level, companies face an *environmental action paradox*, as they need to navigate between addressing biodiversity loss, which is a non-fungible, place-based problem, and addressing all other equally important environmental issues, such as waste management and greenhouse-gas emissions.^91^^,^^92^ In addition to resource constraints creating persistent tensions between possible actions, the different demands for environmental sustainability are also paradoxical, since environmental action in one area can trigger a direct or indirect negative impact on planetary boundaries in other areas. For instance, the adoption of biofuels for corporate vehicle fleets may contribute to the deforestation of old-growth forests through increased demand for agricultural land for biofuel crop cultivation.^93^^,^^94^ Similarly, a company’s transition from fossil fuels to solar energy may require large-scale solar-farm installations. This can increase pressure on productive land with possible leakage effects and can cause direct habitat degradation in areas of biodiversity significance.^95^

By default, companies will apply the same economic logic to this paradox as they do to other business decisions, opting for environmental actions that are least resource-intensive under given resource constraints.^73^^,^^96^^,^^97^ When companies transition from fossil fuels to renewable energy sources, for instance, they may simultaneously be able to reduce emissions and realize cost savings, which makes this environmental action attractive to companies. Biodiversity restoration, meanwhile, requires long-term investments without direct economic returns.^91^ Instead of addressing biodiversity loss, companies may therefore prioritize environmental actions that are easier to quantify and trade, e.g., carbon credits, thereby circumventing substantial changes to their business models with financial instruments.^98^ Companies may also be incentivized by the societal recognition they gain when engaging with highly politicized and mediatized issues, such as climate change, instead of directing their efforts toward biodiversity conservation, which has not yet attracted comparable public attention, despite requiring urgent action.

### Biodiversity action

Lastly, if companies allocate resources to biodiversity at all under their given resource constraints, they need to navigate among different conservation and restoration approaches. This includes choosing between restoration efforts in locations where biodiversity is directly or indirectly impacted by corporate activities, or in areas with no direct connection to the companies’ impact but may be closer to company headquarters or have higher publicity value. Different approaches not only differ in terms of impact but may even conflict with one another due to leakage effects, where biodiversity impacts are displaced from one location to another,^99^ thus creating a *biodiversity action paradox*. For example, if well-intended biodiversity projects in industrialized regions reduce the farmland available for crop production in these countries, unchanged global demand may displace crop production to less industrialized regions with lower environmental standards. If these regions are also global biodiversity hotspots, such displacements may lead to a global-scale net biodiversity decrease. Conversely, efforts to protect unique tropical forests from deforestation may displace land use and biodiversity losses to more industrialized nations, which already have lower levels of biodiversity due to intensive resource use.^100^^,^^101^

Navigating this paradox requires an assessment of the value of different biodiversity losses and gains. This raises complex questions of moral and social equity^102^ as well as questions about the measurability of biodiversity at the science-policy level.^103^ Complementing more established frameworks, e.g., polluter-pays and license-to-operate, the mitigation hierarchy was recently adapted to guide companies’ mitigation of the biodiversity loss caused by their business.^104^ It stipulates that for replacement nature to qualify as offsetting, restoration should take place locally, where the biodiversity loss occurs, and should address the specific biodiversity features that have been harmed and have a positive impact similar in magnitude to the negative impact—the like-for-like principle. This means that deforestation in the Amazonas cannot be offset by ecosystem restoration in the United States, or vice versa. Most importantly, only when all options to avoid or minimize harm are exhausted should offsetting be considered. In addition to its ecological value, biodiversity also carries moral value, which compounds the complexity of its assessment. When destroying biodiversity in one area and displacing biodiversity conservation to another area, the value humans attach to certain nature sites as well as the rights of animals to their habitats is irreversibly lost. Biodiversity offsetting thus also has moral costs that go beyond any net ecological effects that may be obtained.^105^^,^^106^^,^^107^

The economic logic dictates that companies seek to address the paradox by declaring avoidance or minimization unfeasible and using this as a justification for investing in nature restoration projects decoupled from their value chains, sometimes through credit-based compensation schemes without regard to core principles of the mitigation hierarchy, such as the like-for-like principle.^92^ This enables them to do business as usual with little consideration for biodiversity.^108^ Even though credit-based biodiversity offsetting can in some cases deliver the urgently needed biodiversity conservation in the selected target locations, its compensatory nature furthers the financialization and privatization of nature, thus treating biodiversity as a tradable commodity with investment potential. Unless companies are empowered to design initiatives that maximize positive nature impacts while minimizing ecological and moral costs, the economic logic of commodity trading will remain a powerful determinant in how companies navigate this biodiversity action paradox.

## Nested paradoxes constrain biodiversity conservation

Considering business and biodiversity conservation at different levels of the economic system has provided a structured and systematic understanding of how different paradoxes constrain companies’ possibilities for reducing their negative biodiversity impact. As the economic system consists of interrelated sub-systems,^37^ the six paradoxes outlined above form a hierarchically structured system of nested paradoxes, in which responses to higher-level paradoxes constrain the options for addressing lower-level paradoxes. Responses to paradox management at the policy level, such as regulation, taxes, subsidies, and incentives in favor of or against biodiversity conservation or sustainability more broadly limit industry responses to the industry paradox, as they make industry collaborations more or less attractive. These responses, in turn, constrain paradox management strategies at the micro level, as they determine to what extent companies compete on sustainability-related aspects and therefore integrate sustainability into their overall strategies. Further, within the realm of each company, responses to paradoxes at the strategic level are shaped by stakeholder demands and resource constraints. These responses then limit possible responses at the operational level, where the strategic decisions of the company are implemented. Figure 1 provides a representation of the six nested paradoxes, their two poles, and the constraints they impose on lower-level paradoxes, together with possible upward feedback loops.

**Figure 1:**
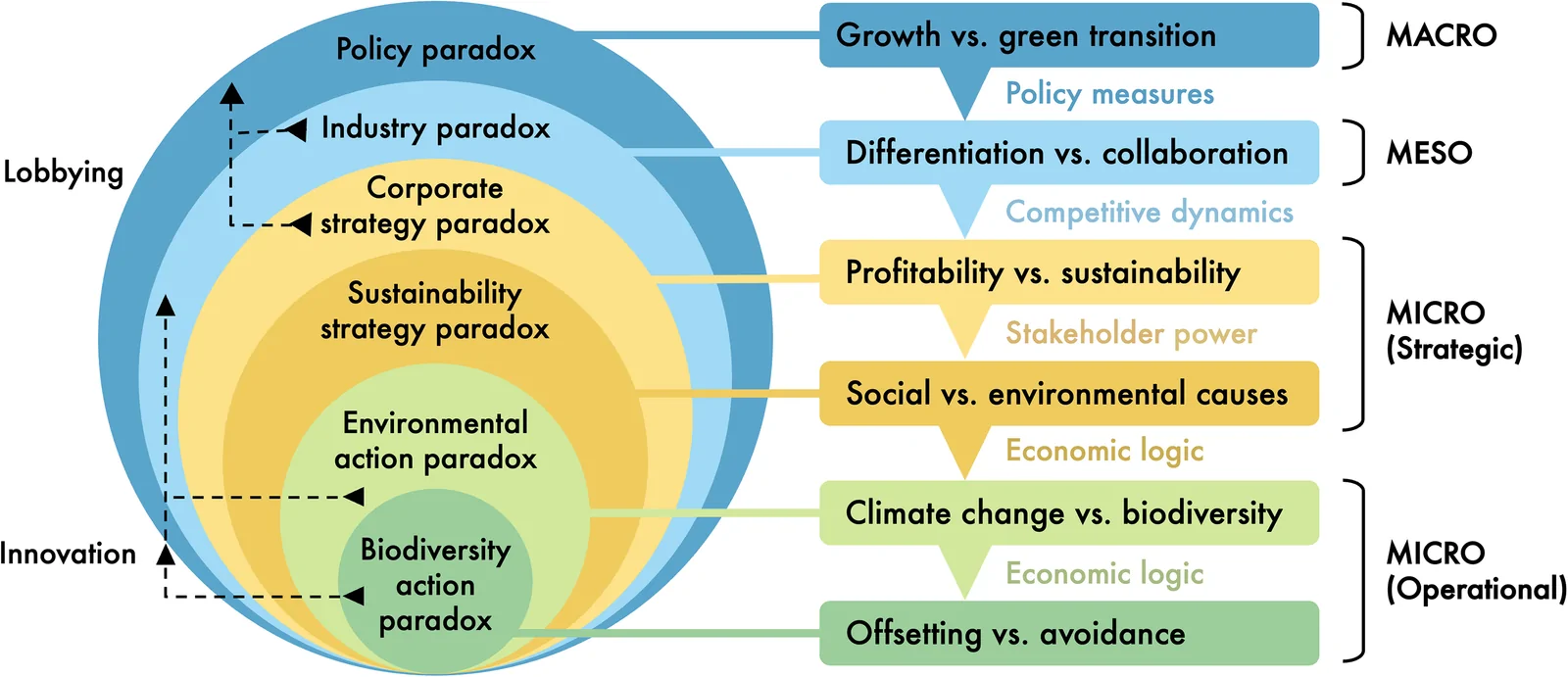
Nested paradoxes around biodiversity conservation The six nested paradoxes shown in the circles consist of two opposing poles each, which are shown in the squares on the right-hand side. The paradoxes start with broader paradoxes around sustainability at the macro (policy) and meso (industry) level, which cascade down to sustainability paradoxes at the company level, which again cascade down to operational paradoxes regarding biodiversity conservation at the company level. Responses to higher-level paradoxes constrain possible responses to lower-level paradoxes in the form of policy measures at the macro level, competitive dynamics at the meso level, stakeholder power at the strategic company level, and the economic logic prevailing at the operational level in companies.

Responses to higher-level paradoxes at the macro and meso level can be influenced by two different upward feedback loops, as depicted in Figure 1. First, corporate innovations can alter both poles of the industry paradox between competition and collaboration, as innovations simultaneously create new areas for collaboration while raising the bar for differentiation. Examples include dissolvable plastics and biodegradable textiles, both of which stimulate collaboration and competition in their respective industries, but do not eliminate the industry paradox as such. Second, both companies and industries can engage in lobbying at the policy level by advocating for strengthening one pole of the policy paradox, i.e., economic growth or sustainability. For example, in response to the EU’s Corporate Sustainability Reporting Directive (CSRD) as well as its proposed scale-back, companies and industry associations lobbied both in favor^109^ and against^110^ these measures.

Box 1 illustrates the nested paradoxes with the fictitious case of a renewable energy company. Figure 2 uses the example from Box 1 to illustrate the nested paradoxes for biodiversity.

Box 1 **Integrative example of the nestedness of the six paradoxes**

RENEW is the renewables division of an established energy company, which develops large-scale solar parks on bought or rented farmland in Northern Europe.

At the **policy level,** governments across Europe are actively supporting the expansion of renewable energy, including large-scale solar parks, as a response to the climate crisis. This support does not come with equivalent requirements to protect or restore biodiversity on the land being developed, as European policymaking is also focused on economic growth and expansion. The broader question of what is lost for nature when farmland is converted to energy production remains largely outside the regulatory frame, thus enabling companies such as RENEW to grow their business without any consideration of biodiversity (policy paradox).

At the **industry level**, the renewable energy sector as a whole pursues the goal of replacing fossil fuels with green energy, which creates opportunities for companies to work together on how this transition is managed. However, the scarcity of suitable land means that RENEW and its industry peers are competing for the same plots of land to erect solar installations (industry paradox).

At the **corporate strategy level**, RENEW seeks to build a profitable business by contributing to the supply of green energy, which is in high demand. At the same time, sustainability concerns around the consequences of solar power installations arise from environmental NGOs and local communities surrounding potential solar parks, as they fear biodiversity loss as well as visual landscape degradation (corporate strategy paradox).

In their **sustainability strategy**, RENEW’s priorities for environmental sustainability in the form of green energy are clear, but come at the expense of social sustainability in the form of local visual landscape degradation and falling property values for local communities in the vicinity of the solar installations (sustainability strategy paradox).

At the **environmental action level**, every solar park that RENEW builds on farmland contributes to reducing carbon emissions and expanding the supply of renewable energy. At the same time, each of these sites occupies land that could otherwise contribute to biodiversity restoration. RENEW’s ability to act on biodiversity is thus constrained by its need to secure new land to expand solar installations (environmental action paradox).

On the sites it develops, RENEW takes **biodiversity action** seriously by retaining existing trees, preserving wet areas and dead wood. The company also invests in local ocean restoration projects, which attract recognition from the local community, even though the company’s business has no direct negative biodiversity impact on marine ecosystems. However, these measures are all responses to damage that has already been decided upon. The result is a biodiversity conservation approach built around making business development acceptable, since the business model precludes or de-prioritizes avoidance (biodiversity action paradox).

**Figure 2:**
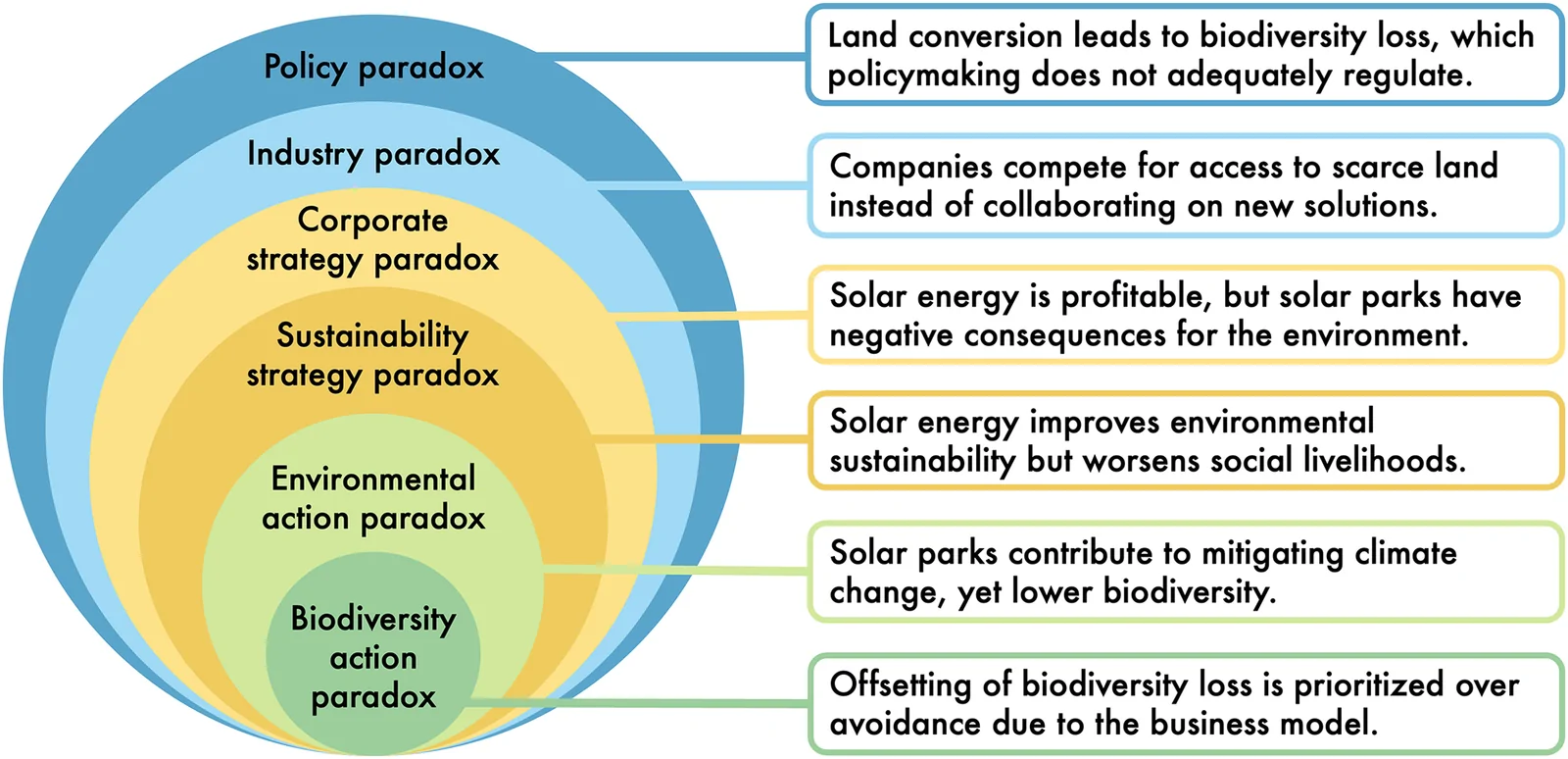
Illustration of the example presented in Box 1 This figure contextualizes the six nested paradoxes, using the fictitious example of an energy company. While it produces green energy, the six nested paradoxes elucidate how it ultimately cannot avoid lowering biodiversity.

Considering the constraints and the paradox management strategies outlined above, current policymaking and standard setting seem to resort to defensive strategies that suppress tensions between oppositional demands or reduce their salience. This evasion of tensions includes adopting the “green economy” discourse (*Denial*) as well as inaction for the sake of economic value creation (*Acceptance*). Further, policymakers address biodiversity and its oppositional demands in compartmentalized spheres (*Spatial Separation*). Examples include the EU Biodiversity Strategy and the EU Competitiveness Compass, both of which are important agendas in their own right but pursue the contradictory goals of biodiversity protection and economic growth, respectively. Lastly, paradoxes at the policy level are managed by adopting ambitious sustainability agendas that postpone problems into the future (*Temporal Separation*), such as the UN Sustainable Development Goals.^111^

Policymaking thus does not sufficiently synthesize the oppositional demands that characterize paradoxes but instead provides opportunities for defensive paradox management at the meso and micro level by prioritizing economic growth and development at the expense of biodiversity conservation at the macro level. At the meso level, this policy approach does not provide sufficient incentives for collaborations among companies, thus leading to defensive paradox management in favor of competition and individual differentiation at the industry level. As illustrated by the nestedness of the six paradoxes (see Figure 1), companies are constrained by the defensive paradox management strategies we see at the macro and meso level. It is therefore unrealistic to expect companies to make massive strides toward biodiversity protection at the strategic or operational level, when industry and policy paradoxes are not addressed with proactive strategies that give sufficient weight to biodiversity conservation.

Accordingly, companies talk the need for action into non-existence by adopting the same “green economy” discourse^112^^,^^113^ as nations and international institutions (*Denial*). They also refrain from substantive biodiversity actions (*Acceptance*) by focusing on environmental issues that generate higher short-term wins than biodiversity, for example, in the form of cost savings, stakeholder consent, or public recognition, such as certain climate actions.^79^^,^^114^ Further, companies circumvent biodiversity mitigation in those places where the loss occurs (*Spatial Separation*) by opting for biodiversity investments in areas not directly affected by their business.^115^^,^^116^ Lastly, companies postpone substantive action into the indefinite future (*Temporal Separation*) by continuously reporting on aspirations, targets, and envisioned solutions while delaying real change.^117^^,^^118^ The paradox perspective thus elucidates why companies approach biodiversity conservation as a resource optimization problem by defensively circumventing industry collaborations, neglecting inconvenient stakeholder demands, seeking tangible benefits from voluntary efforts, or implementing measures that legitimize business as usual.

Without accounting for these nested paradoxes, policy measures risk being too weak to override the constraints companies face from higher-level paradoxes, such as competition or shareholder pressure, which force companies to disregard costly sustainability measures. Rather, effective biodiversity conservation requires that biodiversity is considered at each paradox level by policymakers, entire industries, and corporate decision-makers to get as close as possible to “both-and” approaches in favor of both business and biodiversity. To empower all actors in the economic system to do so, macro-level policy interventions are essential to easing constraints on lower-level paradoxes.

Biodiversity conservation therefore requires robust international governance mechanisms that prevent *Denial, Acceptance*, *Spatial Separation*, or *Temporal Separation* strategies by requiring accountability for both biodiversity loss and protection measures from both governments and companies. At the corporate level, this transparency can stimulate more stakeholder pressure from investors and consumers, thereby increasing reputational risks for companies. Companies can only eliminate such reputational risks by embracing biodiversity conservation as an integral part of their strategies. Further, policymaking should encourage and incentivize companies to embrace the oppositional demands inherent in paradoxes through learning and innovation (*Synthesis*). While corporate innovations can be influential for higher-level paradoxes, they cannot by themselves eliminate the entire hierarchy of nested paradoxes. Rather, policy measures are needed that incentivize companies to synthesize paradoxical demands through innovative “both-and” approaches through industry collaborations in order to remove the competitive potential of innovations.

Successful *Synthesis* approaches to paradoxes necessitate a paradox mindset that seeks to integrate contradictions by expanding the value of resources rather than dividing them between conflicting goals.^24^^,^^119^ In a policy context, a paradox mindset would mean approaching all paradoxes with a holistic, long-term perspective rather than myopically seeking to minimize their salience to achieve short-term economic goals. Policy architectures thus need to be redesigned, moving away from forcing companies to divide resources between the opposing poles of the paradoxes and moving toward expanding the value of resources by incentivizing companies to view biodiversity as an asset, biodiversity loss as a cost, and biodiversity conservation and restoration as an investment in planetary resilience–a prerequisite for long-term economic stability.

The nested-paradox framework can open up avenues for future research on the interrelations between different levels of constraints on company sustainability action, as the natural sciences have only recently begun to pay close attention to the dynamics of corporate decision-making.^3^^,^^114^^,^^118^^,^^120^ Specifically, empirical studies of what macro-level and meso-level actions mean for corporate biodiversity action could be anchored in the paradox levels described in the nested-paradox framework. Examples of future studies include analyses of exceptional *Synthesis* organizations, i.e., those few that have successfully implemented “both-and” approaches at the microlevel despite macrolevel constraints; longitudinal analyses of corporate biodiversity conservation initiatives to better understand defensive temporal and spatial separation responses of companies; or explorations of the cognitive micro-foundations of a paradox mindset for biodiversity conservation. The deliberate integration of biological and management science could strengthen the translational value of biodiversity research and increase its relevance for corporate practice, which is essential to deliver the transformative change of business models and economic systems to halt biodiversity loss, while generating the responsible, long-term value called for by the international community.^4^

In conclusion, the main advantage of paradox thinking over traditional optimization problems is the *Synthesis* approach: where optimization seeks to defensively minimize tensions between opposing poles of the paradox, a synthesis approach embraces these tensions. We argue that paradox thinking is needed not only in policymaking and within companies, but also among actors on financial markets and in educational institutions at all levels, from schooling to professional training, so that the environmental externalities of biodiversity degradation are factored into every decision, rather than sacrificing planetary health for short-term economic gains. By understanding how these nested paradoxes shape decision-making around nature impacts, we are better positioned to regulate, advise, measure, and create effective policy architectures, whether as citizens, policymakers, company owners, managers, employees, or consumers.

## Acknowledgments

The authors acknowledge funding from Aarhus Institute of Advanced Studies 3-2-1-Go Collaborative Theme Group Grant for the project *Bridging Biology & Business: A Cross-Disciplinary Collaboration for Corporate Biodiversity Strategy & Reporting*. J.A.K acknowledges funding from the 10.13039/501100002808Carlsberg Foundation (grant# CF23-0641).

## Author contributions

Conceptualization, I.P., H.A.S., M.S.L., S.S., A.S.B., and J.A.K.; writing – original draft, I.P.; writing – review and editing, I.P., H.A.S., M.S.L., S.S., A.S.B., and J.A.K.

## Declaration of interests

The authors declare no competing interests.

## Declaration of generative AI and AI-assisted technologies in the manuscript preparation process

The authors used *Claude* to generate elements for the graphical abstract. After using this tool/service, the authors reviewed and edited the content as needed and take full responsibility for the content of the publication. The authors declare no other competing interests.
